# Exogenous Methyl Jasmonate Application Improved Physio-Biochemical Attributes, Yield, Quality, and Cadmium Tolerance in Fragrant Rice

**DOI:** 10.3389/fpls.2022.849477

**Published:** 2022-04-25

**Authors:** Adam Sheka Kanu, Umair Ashraf, Lamin R. Mansaray, Farhat Abbas, Sajid Fiaz, Sikandar Amanullah, Christen Shaka Charley, Xiangru Tang

**Affiliations:** ^1^Department of Crop Science and Technology, College of Agriculture, South China Agricultural University, Guangzhou, China; ^2^Sierra Leone Agricultural Research Institute (SLARI)-Rokupr Agricultural Research Centre (RARC), Freetown, Sierra Leone; ^3^Agro-Geo Services (SL) Limited, Freetown, Sierra Leone; ^4^Department of Botany, Division of Science and Technology, University of Education, Lahore, Pakistan; ^5^Institute of Geography and Development Studies, School of Environmental Sciences, N’jala University, Njala, Sierra Leone; ^6^College of Horticulture, South China Agricultural University, Guangzhou, China; ^7^Department of Plant Breeding and Genetics, The University of Haripur, Haripur, Pakistan; ^8^College of Horticulture and Landscape Architecture, Northeast Agricultural University, Harbin, China

**Keywords:** antioxidants, cadmium, yield, quality, fragrant rice

## Abstract

Cadmium (Cd) has detrimental effects on crop plants, whereas, jasmonates (JAs) play a vital role in abiotic stress tolerance in plants. The present study investigated the effects of exogenous application of methyl jasmonate (MeJa) on the physio-biochemical attributes, yield, and quality of two fragrant rice cultivars, i.e., Xiangyaxiangzhan and Meixiangzhan-2 under Cd stress. The experiment was comprised of four treatments, i.e., CK, control (normal conditions); Cd: 100 mg Cd kg^–1^ of soil; MeJa: exogenous application of MeJa at 20 mM; and Cd + MeJa: 100 mg Cd kg^–1^ of soil + exogenous MeJa application at 20 mM. Results depicted that Cd toxicity resulted in a substantial reduction of enzymatic activities and non-enzymatic antioxidants, chlorophyll contents, while enhanced oxidative damage in the terms of lipid peroxidation (higher malondialdehyde (MDA) contents), H_2_O_2_, and electrolyte leakage. Proline contents were found higher whereas protein and soluble sugars were lower under Cd stress as compared with Ck and Cd + MeJa. Exogenous MeJa application further improved the panicles per pot, spikelets per panicle, seed setting (%), 1,000 grain weight, and yield per pot under Cd stress conditions as compared with non-MeJa applied plant under Cd stress. In addition, exogenous MeJa application enhanced the accumulation of macro (N, P, K, Mg, and Ca) and micronutrients (Mn, Zn, Fe, and Cr) in both cultivars under Cd stress, while reduced the Cd contents in different plant parts. Overall, the contents of Cd in different plant organs were recorded as: root > stem > leaves > grains for all treatments. Comparing both cultivars, the grain Cd contents were higher in Meixiangzhan 2 than Xiangyaxianzhan under Cd contaminated conditions. Conclusively, Cd toxicity impaired growth in rice by affecting physio-biochemical attributes, however, Xiangyaxiangzhan performed better than Meixiangzhan-2 cultivar.

## Introduction

Increasing the soil deposition of heavy metals and its associated toxic effects on crop plants has attracted global attention among researchers and farmers (Zaid et al., 2019; Huang J. et al., 2020). Cadmium (Cd) is among the most toxic heavy metals posing threats to crop production, grain quality, and human and animal health (Kanu et al., 2017a; Quddus et al., 2021). Cd is being deposited in arable lands through various sources, such as emission of pollutants from industries, discharge waste from anthropogenic sources, metal smelting, ore mining, and use of synthetic agrochemicals by farmers (Ashraf et al., 2015; Huang and Hartemink, 2020; Huang S. et al., 2020). Cd is known to be easily taken up by plant roots and then translocated to aerial parts, thus, obstructing plant growth and several physiological activities. Cd further interferes with nutrient uptake and its assimilation, photosynthesis, chlorophyll biosynthesis, respiration, and regulates plant signaling mechanism (Anjum et al., 2008; Rao et al., 2019). Moreover, Cd inhibits plant morphological growth, biomass accumulation, yield, and related attributes as well as interferes with plant-nutrient uptake within the rhizosphere in rice (Srivastava et al., 2014). Furthermore, Cd in rice plant primarily disturbs the cellular redox environment and thus causing oxidative stress by the excessive production of reactive oxygen species (ROS) (Farooq et al., 2015). Cd causes cardiac failure, anemia, cancer, hypertension, cerebrovascular infarction, damages the lungs, renal dysfunction in the eyes, osteoporosis, and finally deaths in humans (Cao et al., 2014).

Fragrant rice is famous and desired by consumers due to its special aromatic and cooking characters (Bryant and McClung, 2010). In South-East Asia, it is the most widely desired rice for cultivation among farmers because of its high demand by consumers (Kanu et al., 2017b). The aroma profile is not only necessary to fulfill the consumer demand but also it defend plants from external stimuli (Abbas et al., 2017, 2019, 2020). Therefore, the efforts in producing fragrant rice genotypes with minimum/negligible Cd contamination become eminent especially in a region like South China, where rapid industrial development coupled with less pollution control measures have contributed to the increased heavy metals (HMs) contamination in agricultural lands (Ji et al., 2000). Results from soil survey performed by Zhang and Huang (2000) revealed that a minimum of 13,000 ha of agricultural land in 11 provinces in China were contaminated by varying degrees of Cd, whereas soils in “Zhangtu” and “Sheyang” provinces, that were found to be highly contaminated with HMs in which Cd concentration in both rice grains and soils were above the maximum permissible limits (Huang et al., 2004).

During early grain filling stage in rice, many essential nutrients are remobilized from different plant organs toward grains (Kobayashi et al., 2013). Cd and other mineral elements are generally accumulated in rachises of panicles that form the bases for the termination of vascular bundles in the stem (Fujimaki et al., 2010). Moreover, elements in rachises are then transported to the glumes and developing rice grains through channels and transporters found in the husk around the cells’ embryo and endosperm (Pinto and Ferreira, 2015). The transport rates of ions from rachises to grains were found greatly different among cultivars as well as the type of the element (Sarwar et al., 2010). Therefore, enhancing the Cd inhibition capacity of rice grains based on agronomic practices could provide a promising approach to reduce grain Cd toxicity to ensure food safety.

Jasmonic acid (JA) has been identified to play a significant role as a potential growth regulator which includes the induction of trichome and seed formation, regulation in the reproductive organ development, modulation in seed germination, root growth and development, and regulation in plant responses to various biotic and abiotic stresses (Wasternack and Hause, 2013; Campos et al., 2014). So far, JA application has received much attention due to its multi-functional and stress-alleviatory roles in plants under various abiotic stress conditions (Yan et al., 2010; Ahmad et al., 2015; Kaya and Doganlar, 2016; Ali et al., 2018);

Recent research on MeJa application in Cd mitigation has reported the reduction of chilling injury effects in a number of horticultural crops, such as mango, tomato, fruit, and sweet pepper (Doganlar et al., 2016; Ali et al., 2018) however, the role of MeJa under Cd stress conditions in cereals especially fragrant rice is rarely investigated. The present study was therefore conducted to assess the roles of exogenous JA application on morpho-physiological, yield, and quality aspects of fragrant rice grown under Cd contaminated soil.

## Materials and Methods

### Experimental Site and Conditions

The experiment was conducted in rain protected wirehouse under open-air conditions at the Experimental Research Farm, College of Agriculture, South China Agricultural University, Guangzhou city (23°14′ N, 113°37′ E, 20 M altitude) during the 2017–2018 cropping years. Paddy soil from the research farm was collected to a depth of 20 cm and then air-dried, sieved through a 4 mm sieve, mixed thoroughly, and filled in plastic pots (30 cm height) with 10 kg in each pot. The experimental soil was comprised of 4.96 mg/kg Cd, 18.73 g/kg organic matter, 0.81, 0.9, and 16.79 g/kg total NPK, and 69.15, 10.15, and 109.62 mg/kg, available NPK, respectively, with 5.92 soil pH.

### Crop Husbandry and Treatment Application

The seeds of two fragrant rice cultivars, i.e., “Xiangyaxiangzhan” (Xiangsimiao126 × Xiangyaruanzhan, an indica type of fragrant rice cultivar with a maturity period of about 112–114 days), and “Meixiangzhan-2” (Lemont × Fengaozhan, indica type of fragrant rice cultivar with expected maturity period of 145–147 days), were collected from the Department of Crop Science and Technology, College of Agriculture, South China Agricultural University, Guangzhou, China. The seeds were soaked in a container with deionized water and kept at 25–30°C for 48 h to complete the germination process. Sprouted seeds were then sown in trays containing soil parachute. The trays were placed on leveled paddy field and protected with a plastic sheet in early March to prevent the chilling effect. The 21-day old seedlings were then transplanted to soil containing plastic pots with 3–4 seedlings per hill and 5 hills per pot. Then, 1 week after transplanting, the damaged and affected seedlings were replaced with the healthy ones to get a good stand establishment. Fertilizer was applied with 2.25 g N (urea 46%), 3.33 g P (superphosphate 12% P_2_O_5_), and 1.35 g K (potassium chloride 60% K_2_O) in each pot. Further, 2 weeks before nursery transplantation, Cd in the form of CdCl_2_2.5 H_2_O was added to the soil to obtain the required Cd levels for each treatment in solution form. The experimental treatments were comprised of: Ck, control (normal conditions); Cd: 100 mg Cd kg^–1^ of soil; MeJa: exogenous application of MeJa at 20 mM; and Cd + MeJa: 100 mg Cd kg^–1^ of soil + exogenous MeJa application at 20 mM. The MeJa was uniformly applied two times, i.e., at vegetative and the reproductive stages. The pots were kept in submerged conditions by maintaining a water level of 2–3 cm above the soil surface to create fully anaerobic conditions within the pots.

### Sampling and Measurements

The crop was observed throughout the growth period and plant samples were collected at panicle heading stages and stored at –80°C for physio-biochemical assays while yield components and quality were recorded at the maturity stage after harvesting.

### Photosynthetic Pigments and Physio-Biochemical Assay

Photosynthetic pigments were determined according to Arnon (1949). The fresh leaf samples (0.3 g) at the panicle heading stage were extracted with 15 ml of 95% ethanol and placed in a dark place overnight. The absorbance was read at 665, 649, and 470 nm.

For determining the antioxidant enzymes, the fresh leaf samples (0.3 g) were ground in liquid-N_2_ and homogenized in 6 ml containing 50 mM sodium phosphate buffer (pH 7.8) using mortar and pestle in an ice bath and homogenate were centrifuged at 10,000 *g* for 20 min at 4°C and the supernatant was used to record the enzymatic activities. The activities of superoxide dismutase (SOD) were assayed according to Zhang et al. (2008) following the inhibition of photochemical reduction due to nitro blue tetrazolium (NBT). The SOD activity per unit was defined as the amount of enzyme required to inhibit NBT photochemical reduction to 50% as an activity unit (U). The peroxidase (POD) activity was measured by using the guaiacol method with minor modifications in Lück (1965). The reactions mixture containing 1 ml of sodium phosphate buffer (pH 7.8), 0.2% of 0.95 ml guaiacol, 1 ml of 0.3% H_2_O_2_, and 0.05 ml aliquot of enzyme extract was used and the absorbance was read at 470 nm. Then, 1 unit of POD activity was recorded as the amount of enzyme that caused the decomposition of 1 μg substrate at 470 nm. The catalase activity (CAT) was assayed according to Aebi (1984) whereas 1 unit of enzyme activity (U) was the decomposition of 1 M H_2_O_2_ at A_240_ nm within 1 min in 1 g of fresh leaves samples. The ascorbate peroxidase (APX) activity was estimated by using the “APX determination kit” obtained from Nanjing Jiancheng Bioengineering Institute, China. The manufacturer’s procedure was followed carefully and the absorbance was read at 290 nm. The contents of malondialdehyde (MDA) were determined by following the methods devised by Hodges et al. (1999). Hydrogen peroxide (H_2_O_2_) contents were determined following Velikova et al. (2000). To determine electrical conductivity (EC), the fresh leaf samples were washed with distilled water and then cut into smaller pieces. Leaf disks (0.3 g) were placed in 10 ml of deionized water and incubated at 25°C for 6 h and the (EC1) was recorded with an EC meter (SX650, Sansin, China). Samples were further incubated at 90°C for 2 h, then cool down to 25°C for recording the second EC (EC2). The EC of leaves samples was calculated as: EC (%) = (EC1/EC2) × 100. The protein contents in fresh leaf samples were determined according to Bradford (1976) at panicle heading stage using G-250. The proline contents in fresh leaves samples were determined according to Bates et al. (1973) using ninhydrin. The soluble sugar contents were determined at panicle heading stage following the methods of Zhang et al. (2008). For the reduced glutathione (GSH) and oxidized glutathione (GSSG), estimation was done by using the enzyme-built kits (A006-1 for GSH and A061-2 for GSSG), purchased from the Nanjing Jiancheng Bioengineering Institute, China^1^. The instructions were strictly followed and the absorbance was read at 420 and 412 nm, respectively. Total glutathione was the addition of both GSH and GSSG (GSH + GSSG) contents.

### Grain Yield and Yield Components

At maturity, three pots from each treatment were randomly selected and rice plants from each pot were manually harvested using a sickle and then threshed, the paddy was sun-dried to the required moisture content and grain yield per pot was determined and expressed in grams per pot (g pot^–1^). Total panicles per pot were counted for each treatment and averaged. Filled and unfilled grains were separated and counted manually from each panicle to obtain a total number of filled and unfilled grains per panicle. The 1,000-grain weight was recorded by weighing a random sample of 1,000 filled grains and averaged.

### Grain Quality Attributes

Rice quality attributes were determined from the harvested grains stored at an appropriate moisture content. Brown rice rate was calculated as: (brown rice weight/paddy weight) × 100. The brown rice was then milled using a Jingmi testing rice miller (Zhejiang, China) and the milling recovery was recorded as: (milled rice weight/original weight) × 100. Milling degree was calculated as: (milled rice weight/brown rice weight) × 100. Head rice rate was calculated by separating whole milled grains from 100 grains and head rice rate was calculated on a percentage basis. Grain chalkiness percentage and chalkiness degree were assessed using an SDE-A lightbox (Guangzhou, China). Grain chalkiness is the grains with opaque spots either on the dorsal and or in the center of the grain. Grain moisture, amylose, and protein contents were determined by using an Infratec 1241 (FOSS-TECATOR) grain analyzer.

### Determination of Grain Mineral and Cd Contents in Different Plant Parts

To determine the grain mineral and Cd contents in different plant parts, the plant samples were ground into powder form and digested with HNO_3_ and HClO_4_ in 4:1 (*V/V*) and the contents were estimated by atomic absorption spectrometer (AAS 6300C, Shimadzu, Japan).

### Experimental Design and Statistical Analyses

The research was a two-factor factorial and pots were arranged in a randomized complete block design (RCBD), placing them so as to allow enough space to avoid shading, each treatment had 15 pots for sampling, yield, and quality analysis. The data were analyzed by using the statistical software “Statistix 8” (Analytical Software, Tallahassee, Florida, Unied Satse) while difference among treatments was separated using the least significant difference (LSD) test at *p* < 0.05.

## Results

### Antioxidant Enzymes

Both Cd toxicity and MeJa affected the activities of SOD, POD, CAT, and APX in both rice cultivars. The SOD activity was substantially reduced under Cd toxic conditions for both rice cultivars, however, maximum toxicity was observed in the Cd treatment and SOD activities were reduced when compared with the control treatment for both varieties. In Xiangyaxiangzhan, comparing the control treatment with exogenous MeJa application, 4.85 and 5.92% increase in SOD activities were observed for MeJa and MeJa + Cd treatments, respectively, whereas, 1.1% decrease in SOD activities was observed in the Cd treatment when compared with the control treatment. In Meixiangzhan-2 cultivar, exogenous MeJa application increased SOD activities by 3.85, 30.12, and 37.35% for Cd, MeJa, and MeJa + Cd treatments, respectively. Both Xiangyaxiangzhan and Meixiangzhan-2 cultivars experienced a 2.95 and 14.14% decrease, respectively, in the POD activities in the Cd treatments. For MeJa and MeJa + Cd treatments, 15.38 and 20.07% increase were observed for Xiangyaxiangzhan, respectively, while 3.74 and 38.84% increase were observed for Meixiangzhan-2 cultivar, respectively, when compared with the control treatment. The activities of CAT were decreased by 13.72 and 12.9% for the Cd treatments in both Xiangyaxiangzhan and Meixiangzhan-2 cultivars, respectively, while maximum increased CAT activity (28.41%) was observed in the MeJa + Cd treatment for Xiangyaxiangzhan cultivar. The APX activities saw significant reduction (60.37 and 34.58%) in the Cd treatment for Xiangyaxiangzhan and Meixiangzhan-2 cultivars, respectively; while MeJa addition augmented APX activities in the MeJa + Cd treatment for both cultivars (Table 1).

**TABLE 1:** The effects of exogenous methyl jasmonate (MeJa) application on superoxide dismutase (SOD), peroxidase (POD), catalase (CAT), and ascorbate peroxidase (APX) in two fragrant rice cultivars under cadmium (Cd) stress.

Variety	Treatment	SOD (U min^–1^ g^–1^ FW)	POD (U min^–1^ g^–1^ FW)	CAT (U min^–1^ g^–1^ FW)	APX (U min^–1^ g^–1^ FW)
Xiangyaxiangzhan	CK	371.00 ± 1.00Ab	214.00 ± 0.15Ac	145.47 ± 1.44Ac	0.35 ± 0.02Ab
	Cd	366.93 ± 0.21Ab	207.67 ± 0.02Ad	125.50 ± 0.21Ad	0.14 ± 0.01Ac
	MeJa	389.00 ± 0.52Aa	246.83 ± 1.01Ab	165.60 ± 0.70Ab	0.35 ± 0.02Ab
	MeJa + Cd	393 ± 0.30Aa	256.97 ± 0.17Aa	186.80 ± 0.33Aa	0.64 ± 0.01Aa
Meixiangzhan-2	CK	305.77 ± 0.13Bd	195.67 ± 0.20Bc	165.10 ± 0.49Bb	0.23 ± 0.01Bb
	Cd	317.57 ± 0.23Bc	168.00 ± 0.73Bd	143.80 ± 1.11Bc	0.15 ± 0.72Bc
	MeJa	397.87 ± 0.47Bb	203.00 ± 0.15Bb	172.27 ± 0.65Ba	0.23 ± 0.38Bb
	MeJa + Cd	420.00 ± 0.58Ba	271.67 ± 0.85Ba	174.50 ± 0.82Ba	0.39 ± 0.02Ba

*Three replicated means (± SE) were calculated for each treatment. Values with different letters indicate significant difference between cultivars and treatments at p < 0.05 least significant difference (LSD). CK; control (normal conditions); Cd:100 mg Cd kg^–1^of soil, MeJa: exogenous application of MeJa at 20 mM, and Cd + MeJa: 100 mg Cd kg^–1^of soil + exogenous MeJa application at 20 mM.*

## Chlorophyll Pigments

**FIGURE 1:**
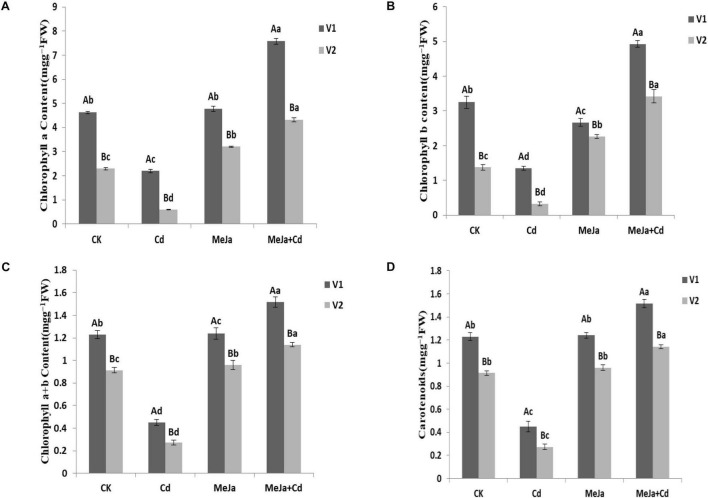
The effects of MeJa on **(A)** Chl a, **(B)** Chl b, **(C)** Chl a + b, and **(D)** carotenoids in two rice cultivars under Cd stress. The values are representative of three replicated means per treatment ± SE. Different letters indicate significant differences between cultivars and treatments at *p* < 0.05, LSD (V1 = Xiangyaxiangzhan and V2 = Meixiangzhan-2).

Cadmium stress significantly affected photosynthetic pigments, i.e., Chl a, Chl b, total chlorophyll contents (Chl a + b), and carotenoids while MeJa supplementation augmented photosynthetic pigments in both cultivars. In comparison with the control treatment, maximum increased Chl a (63.9 and 88.93%), Chl b (51.59 and 146.65%), and total chlorophyll contents (58.81 and 111.36%) were observed in the MeJa + Cd treatment for Xiangyaxiangzhan and Meixiangzhan-2 cultivars, respectively (Figure 1).

### Reduced and Oxidized Glutathione Contents

In the Cd treatments, the levels of GSH, i.e., 20.16 and 15.3% were higher in Xiangyaxiangzhan and Meixiangzhan-2, respectively. Maximum GSSG content (41.7%) was observed in the MeJa + Cd treatment for Meixiangzhan-2 cultivar. Total glutathione (GSH + GSSG) were recorded for the two cultivars studied and the increased levels were higher in Xiangyaxiangzhan than in Meixiangzhan-2 cultivar. The exogenous supplementation of MeJa reduced contents of GSH, GSSG, and total glutathione (GSH + GSSG) in Cd-affected plants.

### Oxidative Damage

The MDA contents in the leaves of both rice cultivars were substantially increased under Cd stress conditions. Exogenous MeJa application and MeJa + Cd reduced the amount of MDA by about 31.8 and 50% for Xiangyaxiangzhan and Meixiangzhan-2 cultivars, respectively. In addition, H_2_O_2_ was found to increase by 45.32 and 35.12% in the Cd treatments for Xiangyaxiangzhan and Meixiangzhan-2 cultivars, respectively. Plants treated with only Cd showed increased H_2_O_2_ content, but decreased significantly with MeJa application. In comparison with the control treatment, a significant reduction in H_2_O_2_ content, i.e., 34.14 and 52.87% was recorded for MeJa + Cd treatments for both Xiangyaxiangzhan and Meixiangzhan-2, respectively. Furthermore, significant reduction of electrolyte leakage (EL) was recorded in plants under MeJa + Cd treatment.

### Proline, Protein, and Soluble Sugar Contents

The proline content increased by 36 and 27% for Xiangyaxiangzhan and Meixiangzhan-2 cultivars, respectively, under Cd stress conditions whereas the proline contents were lowered by 28 and 49% in the MeJa + Cd treatments for Xiangyaxiangzhan and Meixiangzhan-2, respectively. Significant reduction in protein contents, i.e., 14.52 and 38.71% was observed in the MeJa + Cd treatments for Xiangyaxiangzhan and Meixiangzhan-2, respectively. Soluble sugar contents were found to increase almost 36% in the MeJa + Cd for both rice cultivars.

### Yield and Yield Related Components

Cadmium stress reduced the rice yield and its components while an application of MeJa improved yield and its related attributes. In Xiangyaxiangzhan, compared with CK, the maximum number of panicles per pot was recorded in the MeJa + Cd treatment, while the minimum number was recorded in the Cd treatment. Similarly, spikelet number and seed setting rate were reduced under Cd treatment while increased under MeJa and MeJa + Cd treatments. Significant differences were not observed in the 1,000 grain weight in all treatments except for the MeJa + Cd treatment which had a maximum 1,000 grain weight (23.19 g). Furthermore, grain yield was 53.47% higher in MeJa + Cd than Cd treatment. For Meixiangzhan, the highest number of panicles per pot was recorded in MeJa treatment, followed by MeJa + Cd treatment whereas, the least number of panicles per pot were recorded in Cd treatment. In the terms of spikelet number per panicle and 1,000 grains weight, no significant differences were noted except in the MeJa + Cd treatment. Moreover, grain yield in MeJa + Cd treatment was 51.88% higher than Cd treatment. Overall, Cd has negative impacts on grain yield while exogenous MeJa application enhanced the grain yield under Cd stress conditions (Table 2).

**TABLE 2:** The effects of exogenous MeJa application on yield and related components in two fragrant rice cultivars under Cd stress.

Variety	Treatments	Panicles/pot	Spikelet/panicle	Seed Setting (%)	1000 Grain Weight(g)	Yield(g/pot)
Xiangyaxiangzhan	CK	29Ac	112.58Aa	90.61Aa	19.02Bb	75.53Ab
	Cd	26cAd	101.22Ac	76.54Ab	18.23Bb	36.73Ad
	MeJa	35Ab	106.71Ab	92.95Aa	21.56Bb	87.22Aa
	MeJa + Cd	41Aa	98.38Bc	93.23Aa	23.19Aa	56.37Ac
Meixiangzhan-2	CK	27Bb	100.11Ba	86.38Bb	18.81Bc	53.96Bb
	Cd	21Bc	96.00Bab	78.45Bc	18.28Bc	28.91Bd
	MeJa	30Ba	97.55Bab	88.98Bb	20.24Bb	65.46Ba
	MeJa + Cd	29Bab	109.93Aa	92.24Ba	22.25Aa	43.91Bc

*The values are representative of three replicated means per treatment ± SE. Different letters indicate significant differences between cultivars and treatments at p < 0.05, LSD. CK, control (normal conditions); Cd, 100 mg Cd kg^–1^ of soil; MeJa, exogenous application of MeJa at 20 mM; and Cd + MeJa, 100 mg Cd kg^–1^ of soil + exogenous MeJa application at 20 mM.*

### Grain Quality Attributes

Cadmium toxicity was found to affect grain quality traits while significant improvements were observed in quality-related attributes for both rice cultivars augmented with MeJa. For Xiangyaxiangzhan cultivar, brown rice was reduced under Cd treatment by (7.36%) but increased by (5.28%) in MeJa + Cd treatments. Maximum reduction in amylose contents, i.e., 22.71% was recorded for MeJa + Cd treatment, whereas the highest grain chalkiness, i.e., 43.49% was recorded in Cd treatment. Moreover, the maximum reduction in milled and head rice rate, i.e., 20.85 and 21.15%, respectively, was recorded under Cd treatment. Reduced protein content was recorded in the Cd-induced treatment while increased in the treatment with supplemented MeJa.

In Meixiangzhan 2, the Cd was found to have obstructed grain quality while MeJa improved grain quality attributes. For example, protein contents, brown rice rate, and milled rice rate were remained higher under MeJa + Cd than Cd treatment. Moreover, the maximum grain chalkiness (43.49%) and chalkiness degree (27.32%) and minimum brown rice rate (41.54%), head rice rate (52.13%), protein contents (29.39%), and milled rice rate (10.23%) were observed in the Cd treatment. Overall, all grain quality attributes were relatively affected by Cd toxicity and the application of MeJa improved the grain quality of both Xiangyaxiangzhan and Meixiangzhan 2 cultivars (Table 3).

**TABLE 3:** The effects of exogenous MeJa application on grain quality attributes in two fragrant rice cultivars under Cd stress.

Cultivar	Grain Quality Parameters	CK	Cd	MeJa	MeJa + Cd
Xiangyaxiangzhan	Brown rice rate (%)	79.83 ± 0.28Ac	74.36 ± 0.52Ad	82.55 ± 0.44Ab	84.28 ± 0.32Aa
	Milled rice rate (%)	87.45 ± 0.05Ac	72.36 ± 0.13Ad	88.60 ± 0.07Ab	89.59 ± 0.09Aa
	Head rice rate (%)	81.36 ± 0.05Ac	65.53 ± 0.12Ad	83.45 ± 0.10Ab	86.55 ± 0.13Aa
	Chalkiness rate (%)	27.39 ± 0.04Ab	48.47 ± 0.10Aa	23.52 ± 0.13Bc	19.55 ± 0.14Ad
	Chalkiness degree (%)	11.36 ± 0.04Ab	15.6 ± 0.11Aa	10.53 ± 0.12Bc	7.65 ± 0.11Bd
	Moisture contents (%)	10.50 ± 0.03Ab	12.45 ± 0.01Aa	9.59 ± 0.19Bcd	8.94 ± 8.81Bd
	Amylose contents (%)	20.36 ± 0.09Ab	23.64 ± 0.12Aa	19.84 ± 0.03Bc	16.59 ± 0.19Bd
	Protein contents (%)	10.49 ± 0.04Ab	8.58 ± 0.06Ad	9.56 ± 0.05Ac	11.48 ± 0.14Aa
Meixiangzhan 2	Bbrown rice rate (%)	74.75 ± 0.24Bb	52.81 ± 0.26Bd	75.22 ± 0.23Bb	76.16 ± 0.28Ba
	Milled rice rate (%)	76.56 ± 0.15Bb	69.46 ± 0.05Bc	77.00 ± 0.06Ba	76.95 ± 0.03Ba
	Head rice rate (%)	76.87 ± 0.01Bb	50.53 ± 0.12Bd	75.52 ± 0.23Bc	78.49 ± 0.04Ba
	Chalkiness rate (%)	31.68 ± 0.15Bb	54.45 ± 0.14Ba	30.19 ± 0.03Ac	27.61 ± 0.11Ad
	Chalkiness regree (%)	12.66 ± 0.12Bb	17.42 ± 0.02Ba	11.66 ± 0.11Ac	9.97 ± 0.28Ad
	Moisture contents (%)	11.34 ± 0.07Bb	14.44 ± 0.09Ba	10.47 ± 0.03Ac	10.01 ± 0.03Ad
	Amylose contents (%)	21.49 ± 0.03Bb	22.35 ± 6.66Ba	20.04 ± 0.13Ac	19.41 ± 0.29Ad
	Protein contents (%)	9.43 ± 0.07Bb	7.29 ± 0.02Bd	7.92 ± 0.03Bc	9.94 ± 0.03Ba

*The values are representative of three replicated means per treatment ± SE. Different letters indicate significant differences between cultivars and treatments at p < 0.05, LSD. CK; control (normal conditions); Cd:100 mg Cd kg^–1^ of soil, MeJa: exogenous application of MeJa at 20 mM, and Cd + MeJa: 100 mg Cd kg^–1^ of soil + exogenous MeJa application at 20 mM.*

### Grain Mineral Elements

Significant differences were observed in grain mineral contents among treatments. For instance, in Xiangyaxiangzhan, the mineral elements were decreased in the Cd treatment while the application of MeJa enhanced the grain mineral content. Compared with CK, there were 52.32, 40.94, 95.28, 67.45, 67.13, 20.92, 35.98, 35.74, and 62.85% decrease in N, P, K, Mg, Ca, Mn, Zn, Fe, and Cr contents, respectively, in the Cd treatment, while in the MeJa + Cd treatment, the contents of N, P, K, Mg, Ca, Mn, Zn, Fe, and Cr increased by 11.76, 4.74, 11.73, 24.12, 8.53, 8.84,14.53, 16.47, and 41.83%, respectively. Similar trends were observed in Meixiangzhan 2 cultivar, in which Cd induced treatment severely affected grain mineral contents but with MeJa application, grain mineral contents were augmented (Table 4).

**TABLE 4:** The effects of exogenous MeJa application on grain mineral elements (mg/kg) in two fragrant rice cultivars under Cd stress.

		Treatments			
Cultivars	Elements	CK	Cd	MeJa	MeJa + Cd
Xiangyaxiangzhan	N	52.43 ± 0.18Ab	34.42 ± 0.13Ac	53.77 ± 1.49Ab	59.42 ± 0.13Aa
	P	27.33 ± 0.17Ab	19.39 ± 0.04Ad	22.39 ± 0.04Ac	28.69 ± 0.16Aa
	K	2321.3 ± 4.89Ab	1188.7 ± 6.53Ad	2150.1 ± 1.77Ac	2629.9 ± 1.18Aa
	Mg	1246.7 ± 9.40Ac	744.5 ± 5.47Ad	1284.8 ± 3.10Ab	1643 ± 1.61Aa
	Ca	72.74 ± 0.24Ac	43.53 ± 0.15Ad	75.04 ± 0.41Ab	79.53 ± 0.04Aa
	Mn	63.57 ± 0.15Ab	52.57 ± 0.25Ac	63.72 ± 0.32Ab	69.74 ± 0.29Aa
	Zn	30.46 ± 0.04Ac	22.4 ± 0.06Ad	31.86 ± 0.20Ab	35.64 ± 0.10Aa
	Fe	21.95 ± 0.34Ac	16.17 ± 0.25Ad	23.34 ± 0.12Ab	26.28 ± 0.25Aa
	Cr	0.57 ± 0.02Ac	0.35 ± 8.81Ad	0.63 ± 0.02Ab	0.98 ± 0.01Aa
Meixiangzhan 2	N	47.36 ± 0.05Bb	26.52 ± 0.19Bc	47.29 ± 0.08Bb	49.56 ± 0.04Ba
	P	22.54 ± 0.03Bb	15.34 ± 8.81Bc	23.03 ± 0.36Bab	23.18 ± 0.02Ba
	K	2047.6 ± 1.34Bc	1023.8 ± 0.81Bd	2136.1 ± 1.65Bb	2441.9 ± 1.51Ba
	Mg	1111.8 ± 0.29Bd	5414.3 ± 1.16Ba	1133.9 ± 0.94Bc	1285.8 ± 1.43Bb
	Ca	66.16 ± 0.20Bb	32.01 ± 0.40Bc	66.51 ± 0.10Bb	69.43 ± 0.15Ba
	Mn	55.16 ± 0.41Bbc	28.98 ± 0.25Bd	56.08 ± 0.37Bb	57.86 ± 0.24Ba
	Zn	22.35 ± 0.10Bc	17.7 ± 0.32Bd	23.58 ± 0.23Bb	29.74 ± 0.19Ba
	Fe	17.42 ± 0.03Bc	11.08 ± 0.15Bd	18.03 ± 0.14Bb	21.31 ± 0.04Ba
	Cr	0.51 ± 3.33Bc	0.24 ± 0.01Bd	0.56 ± 0.02Bb	0.6 ± 0.01Ba

*The values are representative of three replicated means per treatment. Different letters indicate significant differences between cultivars and treatments at p < 0.05, LSD. CK, control (normal conditions); Cd,100 mg Cd kg^–1^ of soil; MeJa, exogenous application of MeJa at 20 mM; and Cd + MeJa: 100 mg Cd kg^–1^ of soil + exogenous MeJa application at 20 mM.*

### Cadmium Accumulation in Different Plant Parts

Cadmium accumulation in the various organs, i.e., roots, stems, leaves, and grains were substantially higher in Cd treatment, while lower in MeJa applied treatment (Table 2). Exogenous application of MeJa reduced Cd uptake and accumulation in the rice roots and shoots. In the combined treatment (MeJa + Cd), Cd uptake was substantially reduced in the roots, stems, leaves, and grains of Xiangyaxiangzhan by 72.41, 157, 109, and 70%, respectively, while in Meixiangzhan 2, there were 32.75, 119.68, 162.21, and 601.50% decrease in Cd contents of roots, stems, leaves, and grains, respectively. On average, Cd accumulation was in the descending order: root > stem > leaves > grains for all treatments. In summary, Cd uptake and translocation were higher in the roots than stems, leaves, and grains for all the cultivars. However, the grain Cd contents in Xiangyaxiangzhan were substantially lower in Cd + MeJa than Cd treatment whereas found statistically similar (*p* > 0.05) for Meixiangzhan 2.

## Discussion

Recently, the excess application of untreated wastewater and industrial effluents to agricultural lands severely contaminate the soils and build pools of heavy metals. In recent years, the role of jasmonates (JAs) has received considerable attention among researchers due to its involvement in the growth regulation under stress conditions (Welch and Graham, 2002). In this study, we have explored the possible role of exogenous application of MeJa as plant growth regulators on physio-biochemical attributes, oxidative status, yield, and quality traits as well as mineral elements and Cd accumulation in different plant parts of fragrant rice.

The activities of antioxidant enzymes were found reduced under Cd stress conditions while the MeJa application increased antioxidant enzymes activities, i.e., SOD, POD, CAT, and APX in both rice cultivars, though such increment was higher in Xiangyaxiangzhan than Meixiangzhan-2 (Table 1). Similarly, the GSH and GSSG contents were higher in Cd + MeJa than Cd treatment whereas the GSH and GSSG contents were comparatively higher in Xiangyaxiangzhan than Meixiangzhan 2 (Table 5). The increased activity of antioxidant enzymes in response to MeJa application may be attributed to the direct interaction of MeJa with ROS as reported by Chen et al. (2014). In addition, the increased antioxidant enzymes activity due to MeJa supplementation in Cd-stressed plants has been reported by other researchers (Keramat et al., 2009; Yan et al., 2015; Per et al., 2016). Similarly, in *Mentha arvensis* L., the application of plant growth regulators improve the growth, photosynthesis, mineral nutrient, and antioxidant system under cadmium stress (Zaid et al., 2020). Increased SOD, POD, CAT, and APX activity in MeJa supplemented treatments under Cd stress. Strengthening the antioxidant defense system by MeJa supplementation might reduce ROS production and their consequent effects resulting in the alleviation of Cd-induced oxidative stress in Xiangyaxiangzhan than Meixiangzhan-2 rice cultivars. Similarly, the mitigating role of exogenous MeJa application with increased antioxidant enzyme activities under Cd stress conditions has been reported in various plants, i.e., *Solanum nigrum* (Yan et al., 2015), *Brassica juncea* (Per et al., 2016), *Kandelia obovata* (Chen et al., 2014), and *Glycine max* (Keramat et al., 2009). Several previous studies have revealed that MeJa alleviate cadmium stress by regulating ROS detoxification and physio-biochemical damages (Zaid and Mohammad, 2018). In addition, the higher levels of GSH reduced Cd-induced phytotoxic effects and redox regulation and hormonal balancing in *Brassica napus* (Kim et al., 2021) and rice (Kanu et al., 2019) under Cd toxic condition. Furthermore, Cd accumulation led to the substantial reduction in Chl a, b, total Chl, and carotenoid contents with maximum reduction in Meixiangzhan-2 (Figure 1) that may be associated with its sensitivity to Cd stress. The accumulation of Cd in leaves might damage the chlorophyll biosynthesis and thus resulted in reduced chlorophyll contents. In addition, Farooq et al. (2022) reported that Cd stress led to substantial reduction in chlorophyll contents in different rice varieties whereas exogenous MeJa improved the leaf chlorophyll contents (Anjum et al., 2007). Similarly, MeJa application substantially alleviate cadmium toxicity by eliminating the Cd-induced effects on the growth of capsicum (Yan et al., 2013).

**TABLE 5:** The effects of exogenous MeJa application on reduced glutathione (GSH), oxidized glutathione (GSSG), total glutathione (GSH + GSSG), and GSH/GSSG in two fragrant rice cultivars under Cd stress.

Variety	Treatments	GSH (μmolg^–1^FW)	GSSG (μmolg^–1^FW)	GSH + GSSG (μmolg^–1^FW)	GSH/GSSG (μmolg^–1^FW)
Xiangyaxiangzhan	CK	269.63 ± 0.16Ab	68.17 ± 0.44Aa	337.80 ± 0.98Ab	3.96 ± 0.04Ac
	Cd	324.00 ± 0.17Aa	25.36 ± 0.49Ad	349.37 ± 0.72Aa	12.78 ± 0.27Aa
	MeJa	255.70 ± 0.13Ac	55.30 ± 0.81Ab	311.00 ± 1.00Ac	4.62 ± 0.05Ab
	MeJa + Cd	142.37 ± 0.73Ad	45.70 ± 0.85Ac	188.07 ± 0.31Ad	3.12 ± 0.11Ad
Meixiangzhan-2	CK	249.00 ± 0.73Bb	49.87 ± 1.07Bb	298.87 ± 0.74Bb	4.99 ± 0.07Ba
	Cd	287.00 ± 0.52Ba	88.13 ± 0.47Ba	375.13 ± 0.95Ba	3.25 ± 0.06Bc
	MeJa	250.00 ± 0.53Bb	50.90 ± 0.66Bb	300.90 ± 0.17Bb	4.91 ± 0.03Ba
	MeJa + Cd	127.00 ± 0.31Bc	29.06 ± 0.97Bc	156.07 ± 0.14Bc	4.38 ± 0.09Bb

*The values are representative of three replicated means per treatment ± SE. Different letters indicate significant differences between cultivars and treatments at p < 0.05, LSD. CK, control (normal conditions); Cd,100 mg Cd kg^–1^ of soil; MeJa, exogenous application of MeJa at 20 mM; and Cd + MeJa, 100 mg Cd kg^–1^ of soil + exogenous MeJa application at 20 mM.*

**FIGURE 2:**
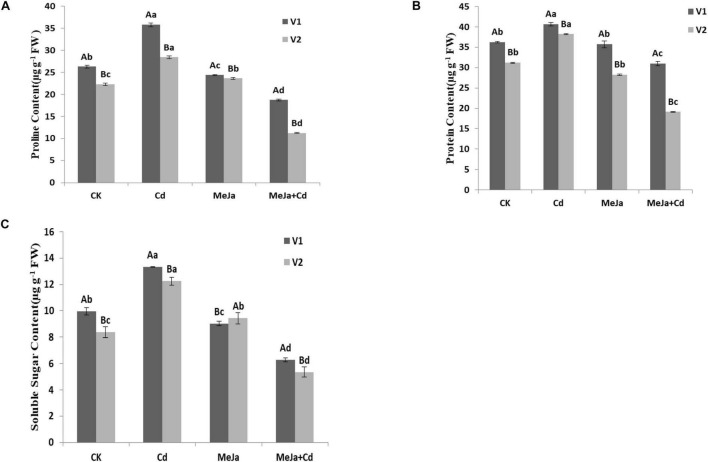
The effects of MeJa on **(A)** proline, **(B)** protein, and **(C)** soluble sugars in two rice cultivars under Cd stress. The values are representative of three replicated means per treatment ± SE. Different letters indicate significant differences between cultivars and treatments at *p* < 0.05, LSD (V1 = Xiangyaxiangzhan and V2 = Meixiangzhan-2).

In our study, the increased production of MDA, H_2_O_2_, and EL were observed in both cultivars under Cd stress, however, exogenous MeJa provided relieve against oxidative stress (Table 6). Moreover, free proline, protein, and soluble sugar contents in both rice cultivars were found higher in Cd treatments (Figure 2). In plants generally, proline directly act as an antioxidant providing protection for the cell against free radical damages for enabling a more favorable environment to reduce Cd sequestration (Shah and Nongkynrih, 2007). In addition, the yield and related traits were substantially reduced under Cd toxic conditions whereas foliar MeJa application improved the panicles/pot, spikelet/panicle, seed setting (%), 1,000-grain weight, and grain yield (Table 2). Higher grain yield under MeJa treatment might be associated with the improved yield related attributes of both rice cultivars. Substantial reduction in yield and other agronomic attributes were noticed in Cd exposed rice plants (Majumdar et al., 2020), whereas MeJa alleviate the toxic effects of Cd by modulating the signaling mechanism in rice (Verma et al., 2020). Our findings corroborate with White and Broadley (2005), who concluded that exogenous application of plant growth regulators increased rice growth and yield under Cd stress conditions. Rice grain quality were affected by Cd toxicity and the exogenous application of MeJa improved grain quality attributes. External factors affecting plant led to significant changes in rice quality attributes especially aromatic rice. Reductions in rice quality attributes (brown and milled rice rates as well as head milled rice rate) were found in both rice cultivars under Cd toxicity whereas MeJa supplementation improved the grain quality characters (Table 3). Similarly, Ashraf et al. (2017) and Ashraf and Tang (2017) reported the deterioration in rice grain quality attributes under heavy metal toxicity. It has been demonstrated that exogenous hormone application improves plant fitness *via* maximizing the transcriptional level of related biosynthesis pathway genes in plants (Abbas et al., 2021a,b). Mineral elements contents were increased in treatments with applied MeJa, while under Cd stressed treatments, mineral elements contents reduced greatly. Rice plants require macro- and micronutrients (K, Ca, Mg, Fe, Cu, Mn, Ni, and Zn) for the growth, development, and increased productivity (Pinto and Ferreira, 2015). Various forms of interactions among plants exist during the uptake and accumulation of mineral elements (Du et al., 2013; Pinto and Ferreira, 2015), since Cd compete with important mineral nutrients uptake by plant as reported by Nazar et al. (2012) and Asgher et al. (2014). The present study revealed that the Cd accumulation in rice plants considerably reduced the uptake of essential mineral nutrients (Table 4) whereas MeJa application mitigated such competition by restoring the root-to-shoot translocation mechanism of key mineral elements. Similar observations have been reported by Gómez et al. (2010) for *Solanum lycopersicum* and Kováčik et al. (2011) for *Scenedesmus quadricauda*. Furthermore, phloem sap in rachis forms the closest source for supplying elements to stimulate grains development, therefore, the concentration of Cd and other elements between rachises and grains shows the extent of grains ability to accumulate different elements. Our study revealed genotypic differences in mineral elements accumulation in grains. Additionally, Cai et al. (2010) revealed that exogenous application of growth regulator increased certain mineral nutrient uptake, i.e., Zn, Cu, and Mn, while Cd uptake was decreased in rice in concentration and a genotype-dependent manner. Previous studies have shown that many quantitative trait loci (QTLs) aiding the mineral accumulation in rice grains exist and are largely dependent on the environment (Du et al., 2013). Hence, rice cultivated under Cd stress conditions continuously faces a reduction in the accumulation of grain mineral elements. Furthermore, genes and QTLs for mineral elements accumulation have been reported to mediate transporting several elements at the same time (Gill et al., 2013). Improvements in rice yields and grain micro-nutrients’ accumulation might be the result of plants higher nutrients absorption ability from the soils enriched with a suitable growth regulator, hence, increased the nutrient use efficiencies (Farooq et al., 2015).

**TABLE 6:** The effects of exogenous MeJa application on malondialdehyde (MDA), H_2_O_2_, and electrolyte leakage (EL) in two fragrant rice cultivars under Cd stress.

Variety	Treatments	MDA (μmolg^–1^ FW)	H_2_O_2_ (μmolg^–1^ FW)	EL (%)
Xiangyaxiangzhan	CK	5.77 ± 0.19Bb	16.40 ± 0.15Bb	32.55 ± 0.84Bb
	Cd	10.47 ± 0.17Ba	23.83 ± 0.12Ba	58.43 ± 0.29Ba
	MeJa	6.06 ± 0.08Bb	16.77 ± 0.16Bb	32.13 ± 0.46Bb
	MeJa + Cd	3.93 ± 0.12Bc	10.80 ± 0.15Bc	21.40 ± 0.21Bc
Meixiangzhan-2	CK	7.80 ± 0.11Ab	19.16 ± 0.08Ab	43.91 ± 0.26Ab
	Cd	12.86 ± 0.08Aa	25.90 ± 0.12Aa	63.27 ± 0.15Aa
	MeJa	7.27 ± 0.11Ac	18 ± 0.26Ab	38.59 ± 0.54Ac
	MeJa + Cd	3.90 ± 0.12Ad	9.03 ± 0.61Ac	24.46 ± 0.17Ad

*The values are representative of three replicated means per treatment ± SE. Different letters indicate significant differences between cultivars and treatments at p < 0.05, LSD. CK, control (normal conditions); Cd,100 mg Cd kg^–1^ of soil; MeJa, exogenous application of MeJa at 20 mM; and Cd + MeJa, 100 mg Cd kg^–1^ of soil + exogenous MeJa application at 20 mM.*

Substantial Cd contents were noticed in different plant parts, i.e., root, stem, leaves, and grains under Cd treatment, however, the relative Cd contents were lower under MeJa treatment (Table 7). Rice plants treated with MeJa had less Cd in all plants part as MeJa mediates in binding Cd into other complexes which cannot be easily taken up by the plants. On the other hand, Cd uptake is found to be largely influenced by rice genotypic character for metal accumulation and speciation in different plant parts. In this study, genotypic differences existed in Cd accumulation with Xiangyaxiangzhan cultivar accumulating less Cd in different plant parts compared with Meixiangzhan-2 under the same conditions and agronomic practices. Further studies are recommended to further explore the understanding of genotypic variations regarding Cd accumulation in rice since it is very difficult to select inheritable low-heavy-metal accumulation traits in rice plants as suggested by Tan and Barrington (2005).

**TABLE 7:** The effects of exogenous MeJa application on Cd contents in different plant parts (μg/g dry weight) in two fragrant rice cultivars under Cd stress.

Cultivar	Treatments	Roots	Stems	Leaves	Grains
Xiangyaxiangzhan	CK	3.5 ± 0.03Bb	0.74 ± 0.02Bc	0.23 ± 0.01Bc	0.01 ± 3.52Bc
	Cd	369.41 ± 1.18Ba	16.36 ± 0.04Ba	4.27 ± 0.05Ba	1.15 ± 0.19Ba
	MeJa	2.55 ± 0.12Bb	0.53 ± 0.01Bc	0.18 ± 0.02Bc	0.01 ± 3.00Bc
	MeJa + Cd	352.03 ± 0.04Ba	10.29 ± 0.01Bb	3.11 ± 5.77Bb	0.51 ± 5.77Bb
Meixiangzhan 2	CK	4.62 ± 2.47Ab	0.96 ± 0.18Ab	0.32 ± 0.01Ab	0.09 ± 0.05Ab
	Cd	471.47 ± 0.07Aa	34.59 ± 0.06Aa	3.56 ± 0.01Aa	2.88 ± 0.01Aa
	MeJa	4.04 ± 0.11Ab	0.88 ± 0.06Ab	0.33 ± 0.32Ab	0.06 ± 3.33Ab
	MeJa + Cd	473.48 ± 0.11Aa	31.43 ± 0.06Aa	3.12 ± 0.01Aa	2.01 ± 3.33Aa

*The values are representative of three replicated means per treatment ± SE. Different letters indicate significant differences between cultivars and treatments at p < 0.05, LSD.*

## Conclusion

Cadmium toxicity has negative consequences on physio-biochemical attributes, rice yield, grain quality, and mineral nutrient accumulation in grains whereas exogenous MeJa improved the overall performance of both rice cultivars. MeJa application led to improved anti-oxidant activity, chlorophyll contents, while reduced oxidative damage under Cd stress conditions. Moreover, rice roots can accumulate higher Cd than shoots, however, transport in above ground plant parts may vary among cultivars. Overall, Xiangyaxiangzhan variety performed better than Meixiangzhan 2 under Cd toxic conditions. Further studies are needed to examine the involvement of MeJa in Cd stress tolerance and regulation mechanism in fragrant rice.

## Data Availability Statement

The original contributions presented in the study are included in the article/supplementary material, further inquiries can be directed to the corresponding author/s.

## Author Contributions

AK and UA designed and performed experiment. AK, UA, LM, and FA perform the experiments. SF, SA and CC help in formal analysis. AK, UA, FA, and XT drafted the manuscript. All authors approved the final version of the manuscript.

## Conflict of Interest

AK and LM were employed by Agro-Geo Services (SL) Limited. The remaining authors declare that the research was conducted in the absence of any commercial or financial relationships that could be construed as a potential conflict of interest.

## Publisher’s Note

All claims expressed in this article are solely those of the authors and do not necessarily represent those of their affiliated organizations, or those of the publisher, the editors and the reviewers. Any product that may be evaluated in this article, or claim that may be made by its manufacturer, is not guaranteed or endorsed by the publisher.

## Footnotes

1
http://www.njjcbio.com
